# Exploring sustainable care pathways - a scoping review

**DOI:** 10.1186/s12913-022-08863-w

**Published:** 2022-12-30

**Authors:** Eva Walderhaug Sather, Valentina Cabral Iversen, Marit Folsvik Svindseth, Paul Crawford, Frøydis Vasset

**Affiliations:** 1 Faculty of Medicine and Health Sciences, Department of Health Sciences, The Norwegian University of Science and Technology (NTNU), Trondheim, Norway; 2 Faculty of Medicine and Health Sciences, University of Nottingham, Nottingham, UK; 3 Department for Health and Social Sciences, University College in Molde, Molde, Norway

**Keywords:** Care pathways, psyciatric services, patient transition, community mental health

## Abstract

**Background:**

Patients with mental health problems experience numerous transitions into and out of hospital.

**Aim:**

The review studies assessing clinical care pathways between psychiatric hospitalization and community health services.

**Methods:**

We used publications between 2009–2020 to allow a broad scoping review of the published research. Sixteen review-articles were identified, 12 primary studies were chosen, both on care pathways in the transition between psychiatric hospital and community.

**Results:**

Organizational issues: Systems and procedures to ensure clear responsibilities and transparency at each stage of the pathways of care. Resources: Information-technology in objectively improving patient outcome. Information/documentation: Providing patients with adequate structured information and documented plans at the appropriate time. Patient/families: Continuous collaborative decision-making. Clinical care and teamwork: Collaboration between mental health and other professionals to guarantee that planned activities meet patient need. Ethical issues: Respectful communication and patient-centred, non-humiliating care.

**Conclusions:**

System and procedures ensure clear responsibilities and transparency. Information technology support decision-making and referral and objectively improve patient outcomes in care pathways. Collaboration between mental health and other professionals guarantee that planned activities meet patients’ needs along with regular meetings sharing key information. Around-the-clock ambulant-teams important to transition success. Informed-shared decision-making between parties, support patient participation and respectful communication.

**Supplementary Information:**

The online version contains supplementary material available at 10.1186/s12913-022-08863-w.

## Introduction

Care pathways are used increasingly worldwide to organize patient care. However, there are different views about their effectiveness, outcomes, and impacts [1, 2]. There is a growing interest in extending care pathways in both primary and mental health care to improve the quality of service through enhanced care coordination [3]. Vanhaecht et al. [1, 2] defined the term ‘care pathway’ as follows: ‘A care pathway is a complex intervention for the mutual decision making and organization of care processes for a well-defined group of patients during a well-defined period’.

It seems to be consensus on the importance of early intervention in the treatment of mentally ill patients [1, 2]. Rutman et al. [4], find that the evidence between the relationship between care pathways and care coordination are sparse.

Care pathways are a way to improve care coordination and operationalise the patient-focused care concept [5]. The aim of care pathways is to improve outcomes by providing a mechanism to coordinate care and reduce fragmentation and, ultimately, costs [6]. However, several studies have revealed that care pathways improve the components of care coordination [7–9].

Care pathways are not simple or straightforward but rather complex interventions; they comprise separate elements that seem to be essential to the proper functioning of the intervention; they target multiple outcomes and involve multiple interventions, and the ‘active component’ is difficult to specify [10, 11]. Consistent with this definition, the characteristics of care pathways include ‘an explicit statement of the goals and key elements based on evidence, best practice, and patients’ expectations and their characteristics’. This includes a range of elements: facilitation of communication among team members, patients, and families; coordination of the care process by coordinating the roles and activities of interprofessional care teams, patients, and their relatives; documentation, monitoring, and evaluation of variance and outcomes; and identification of the appropriate resources [10].

Care pathways are popular tools to improve the quality of the diagnostics, treatment, and follow-up of hospital patients [10, 12]. Mental healthcare delivery systems and policies have shifted the focus from more extensive psychiatric inpatient settings to community services [3, 13, 14]. Yet mental health in-patient facilities continue to discharge patients too early, without clear discharge planning, and the post discharge follow-ups do not function properly [15].

People with mental illness often experience stigma and social rejection when returning to their communities [16]. Research reports engagement in 6–12 months community rehabilitation compared to shorter inpatient rehabilitation [17]. Transition of care is particularly important for patients with mental health problems who experience numerous transitions into and out of hospital. This transition comprises hospital discharge, post-discharge support at the next level/location of care and the engagement of the patient and caregiver in these processes [18].

Patients as high-frequency users of psychiatric services in hospital, remaining well for only short periods of time [19]. These patients have diverse preferences for care and face a variety of barriers associated with mental health treatment [19, 20].

Many persons with mental health problems will have need of services from different levels at the same time and this context suggest need for easy access to a range of treatment and providers [3, 21–23]. Essential elements in this care process are open communication and well-organized, delegated coordinating roles for interprofessional care teams [24, 25]. With care pathways, high-performance teams can be built [4]. Chew-Grahee [26] pointed out that, depending on its quality, communication could function as both a promoting factor and a barrier to success. Starfield et al. [27], identified the following key elements in the integrative functions of primary care: first contact, continuous, comprehensive care and coordinated care. These four elements are implicitly incorporated into the health care system to improve outcomes [28]. Vickers et al. [29], noted that expanding integrated mental health care in the primary care setting/services resulted in increased staff and provider satisfaction.

Thus, the aim of a care pathway is to enhance the quality of care across the continuum by improving risk-adjusted patient outcomes, promoting patient safety, increasing patient satisfaction, and optimizing the use of resources [11, 30, 31].

The extent to which these discourses have impacted on individual clinical practice and care experiences remains unclear. Crucially, the involvement of patients at points of transfer of care from the community to inpatient settings and back to the community has been underreported. Tveiten et al. [32], advised giving patients in mental health contexts a voice to express their concerns and have them addressed. A study about patients’ knowledge and the power imbalance in the doctor–patient relationship supports our assertions that patients need knowledge and power to participate in shared decision-making processes [33].

The study offered several recommendations for enhancing patients’ participation by simplifying the trialled pathway and the accompanying guidelines and strategies to improve communication between nurses and general practitioners. Pelto-Piro et al. [34], found that paternalism still clearly appears to be the dominant perspective among staff caring for patients in psychiatric inpatient care settings. Grim et al. [35], described obstacles to legitimizing patient knowledge, including relational issues that patients highlighted: being independent, often being dismissed and unable to edit their testimonies. Health providers typically described workflow issues, patients’ insufficient decision-making competence and patients’ vulnerability to stress factors.

Care Pathways are developed and implemented across the international health care arena, evidence to support their use has been equivocal, and the understanding of their ‘active ingredients’ is poor. Care pathways are ‘complex interventions’ and are increasingly being implemented for a variety of purposes in a range of organizational contexts [1, 2, 36]. The development and implementation of care pathways are based on interprofessional teamwork, an understanding of the practical organization of care and the integration of a set of evidence-based key interventions [1, 2]. A recent study evaluating the implementation of a transitional discharge model, an intervention for community integration of clients with mental health illness, concluded that this implementation provides cost-effective supports to help keep clients in the community and out of hospital [37]. And as reported previously [22] the establishment of relationships among patients, inpatient staff, and community staff is of utmost importance in the transition process between inpatient and community mental health care.

In mental health care pathways involve a significant change work which should give the patients more holistic and coherent services but are hampered by professionals experiencing large workload, frustration and stress associated with registration, and administration systems [38]. A bottom-up implementation strategy for implementing care pathways is recommended [39]. Ruben von Zelm [40] concluded that the implementation and normalization of a care pathway depends on the following: involved professionals, including physicians, to achieve the desired outcomes; understanding and appreciation of the content, goals; and related standardization of the care pathway and play an active role in the improvement team. Implementing the pathway requires: resources, including a care pathway facilitator and a clinical data system; the improvement team’s experience, collaboration, and clinical leadership; and individual experience and expertise [12].

The purpose of the study is to provide an overview of studies assessing clinical care pathways for people with mental health problem in the transitional process from hospital to the community.

## Methods

Grant and Booth [41] described scoping review as ‘a preliminary assessment of potential size and scope of available research literature.’ We followed Arksey and O’Malley’s approach [42], and used Levac [43] as a guide, for how to operationalize each step.’

Arksey and O`Malley`s [42] five-stage methodological framework including:

1) identify the research question; 2) identify relevant studies/articles in a literature search; 3) select relevant studies/articles based on inclusion and exclusion criteria; 4) chart the data extraction in a standardized form; and finally, 5) collate, and summarize the results, including the assessment of methodological quality.

A scoping review method was chosen to gain a comprehensive overview of the literature to map key concepts, identify knowledge gaps and convey the breadth and depth of the field [44–46]. At the abstract screening stage, we keep the references that meet the inclusion criteria for review and those that we are unsure about. We dismissed the rest. The references we kept was moved to the next stage in the selection process: Arksey and O`Malley [42] full-text screening.

### Inclusion and exclusion criteria

The study population included adult individuals (people who are 18 years of age or older). We excluded studies which children were involved. Care pathways for specific mental health diagnoses were not searched for but included if they fitted the overall purpose of the study. We limited the search to 2009 – 2020 to allow a scoping search of the published research. We excluded editorials and discussion papers, and research protocols.

Disagreements regarding inclusion or exclusion were resolved through discussion with two other researchers.

### Search strategy

#### Stage 1: Identify research questions

First step was to identify the research. The research questions are presented as above. The purpose of the study is to provide an overview of studies assessing clinical care pathways for people with mental health problem in the transitional process from hospital to the community.

#### Stage 2: Identify relevant studies/articles

To ensure the identification of relevant literature, an experienced librarian critically reviewed the search strategies, search terms and inclusion and exclusion criteria. These searches included studies published in English between 2009 and 2020. The following scientific electronic databases with keywords were systematically searched: ProQuest/Health & Medicine, CINAHL Complete, Cochrane trials and Cochrane reviews, PsychoInfo, Medline, PubMed, and Google Scholar.

The following search terms were included to represent care pathways in mental health in transition from hospital to the community (Medical Subject Headings (MeSH) terms used for searches in Medline are marked with an*): ‘care pathways*, ‘integrated care pathways*, ‘critical pathways’, ‘clinical pathways’, ‘mental health*, ‘adults’, combined with ‘transitions from hospital to community’, ‘referral’, ‘discharge*,’care planning*, ‘coordinating’, ‘hospital and mental health services.

We reviewed the reference lists of studies identified– especially systematic reviews and traditional literature reviews–and included relevant studies in the scoping exercise. Key journals were also hand-searched to identify articles that may have fell outside the database and reference list searches. We did hand-search in key-journals, especially in articles in Norwegian Journals, that could not be accessed in full text in English. This were excluded.

#### Stage 3: Selection of relevant studies based on inclusion and exclusion criteria.

**Fig. 1:**
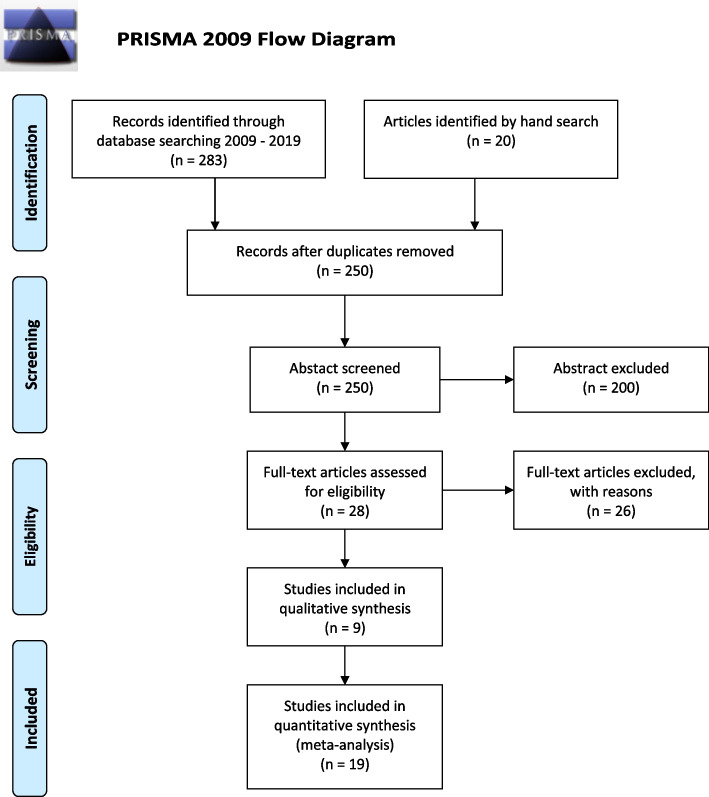
Flow-diagram presents a complete overview of the inclusion and exclusion criteria

This stage entailed the study selection process as illustrated in a Prisma flow diagram (Fig. 1). The 283 articles in the scoping review resulted in 28 eligible full text articles [44, 46].

A total of 250 records were identified by the librarian conducting the systematic literature search and were imported into refence management software. After the removal of 200 abstracts, 28 full-text articles were assessed for eligibility, and then full-text articles excluded with reasons, we had at last 28 studies; studies included in qualitative synthesis 9 and studies included in quantitative synthesis 19, see PRISMA 2009 Flow Diagram, Fig. 1. Two authors (EWS and MFS reviewed the same first, and then FV and VCI reviewed the half (of selected) 200. Disagreement regarding inclusion or exclusion were revolved through discussion. Four authors (EWS, MFS, FV, VCI) read the full text of the remaining 26 records and assessed their eligibility. The four authors conducted meetings to discuss their assessments of the abstracts and full-text articles and the inclusion and exclusion criteria to reach agreement on the studies to be included.

The study population included adults’ individuals; people who are 18 years of age or older. We excluded studies which children were involved.

To meet the overall purpose of the scoping review, the included 28 studies focused on care pathways in transition between psychiatric hospital and the community mental health services.

#### Stage 4: Charting the data

We extracted and coded each eligible and included article according to the following descriptive content of the 28 selected studies: 19 quantitative synthesis and nine qualitative syntheses. The descriptive data comprised authors, country of origin, aims, data collection and measurements, study sample and results. The extraction and charting of the data were conducted by author with input from two other researchers. Descriptive content of the selected review studies is presented in Table 1 and Table 2 and Table 3.

**Table 1:** Characteristics of review articles

Author and country of origin	Aims	Study design	Data Collection and measurments	Study sample	Results
1: Allan et al., (2020). UK	To synthesize the available literature on pathways of care in At Risk Mental States or prodromal psychosis, an investigate the barriers and facilitators to receiving care for ARMS	Included English studies publish between 1985 and 2019, and the study described or related to pathways to care	The CINAHL Complete, EMBASE, Medline Complete, PsycINFO and PubMed databases were searched	Reported data came exclusively from an ARMS population	Mental health professionals, and general practitioners played a key role in help seeking. Family involvement was also found to be an important factor
2: Allen et al., (2009).UK	Identify the effectiveness of care pathways in mental health settings presented in a narrative way	Systematic review of seven randomized controlled trials published 1980–2008. Using flow chart and RCT method	Using Medline, CINAHL, Cochrane. Use three stages strategy and three stages filtering process	Seven studies from different countries with adult and child in mental health. English, Germany, and French translation was available	Care pathways are most effective in patients with predictable managing of mental symptoms to support proactive management and is more uncertain when including patients with more uncertainty in behavior. Pathways of care could also improve documentation, communication, and change professionals´ behavior in desired direction
3: Amaral et al., (2018). Brazil	Explorative systematic review to highlight evidence for each pathway stage	The review synthesized narrative for the 25 studies ranging from 1999 to 2017	LILACS; MEDLINE; SCIELO databases used to searched for paper. 25 studies included 9 quantitative and 14 qualitative and two mixed methods	In all 25 studies were from Brazil. Both patients and health personnel views were included	Complex social networks were involved in the studies and the points of first contact varied. A high proportion of patients is treated in specialized services and there is a stressing lack of integration between emergency, hospital and community
4: Anderson et al., (2010). Canada	Examine the associations between pathways of care and sex, socio economic and ethnical determinants of pathway, and duration of untreated psychosis as well as help-seeking behavior	Systematic review with inclusion of 30 papers from 1985 and 2009 in 16 countries, with both quantitative and qualitative design	Using Medline, HealthStar, EMBASE, PsycINFO databases. Then manual search in 15 journals	In all 30 studies from Australia, Asia, USA, Africa, Europa. Both patients, relatives and health personnel views were included	Found that the first contact for patients was a physician, but the referral source was emergency services. They did not find consistent results across the studies that explored the sex, socio-economic, and ethnic determinants of the pathway. More research needed to understand disparities between pathways of care and which factors that could increase patients help-seeking behavior
5: Chen et al., (2015). Canada	Evidence to improve quality and efficiency of care for patients with schizophrenia	Scoping review methods following PRISMA guidelines. Metanalysis not being done due to differences in methods in the papers	Paper from MEDLINE, PsycINFO, Health Star, EBM Review Cochrane Database. 7 focused on describing process-related data and 6 examined clinical outcomes	In all 13 papers (Hong Kong, Germany, UK) were included from 1998 to 2014. Inclusion criteria narrowed to people suffering from. schizophrenia. Both patients and health personnel views were included	Finings in three areas: Content, development, effectiveness. Pathways of care show promising results in increasing quality and efficiency for patients with a diagnosis of schizophrenia, but more evidence is needed
6: Deneckere et al. (2012). Belgium	Explore the relationship between effectiveness and how care pathways promote teamwork	Systematic review with inclusion of 26 relevant studies	Systematic literature search strategy in three electronic databases – MEDLINE, Embase, and CINAHL, combined with Mesh and non-Mesh terms for care pathways and teamwork	In all 26 studies used the result of an international expert panel on team indicators in care processes to identify search terms	That was frequently mentioned the need for a multidisciplinary approach and educational training sessions. Necessary conditions are a context that support teamwork and to achieve these, each care pathways requires a clearly defined team approach customized to the individual teams’needs
7: Doody et al. (2017). Ireland	Identify families’ experiences of care planning involvement in adult mental health services	Review was guided by a framework which is a methodological approach allowing for concurrent synthesis of qualitative and quantitative research methods	An integrative literature review where electronic databases and grey literature were searched for papers published between 2005 and 2016 from CINAHL, Scopus, Web Science, PsyInfo, MEDLINE, PsyArticles	In all 15 papers from UK, USA, Sweden, Norway, Italy, Israel met the inclusion criteria Thematic analysis generated three themes	1: Families’ experiences and collaboration, 2: families’ perceptions of professionals and 3: families’ impressions of the care planning process. Collaborative decision-making was not regularly experienced by families, lack of communication, confidentiality constraints and a claim of ‘insider knowledge’ of service users. Care planning were perceived to be uncoordinated and their lived experiences not always appreciated
8: Durbin et al. (2012). Canada	To get evidence on the quality of information transfer between primary care physician (PCP/GP) and specialist mental health providers for referral requests and after inpatient discharge	A scoping interview of the literature was conducted to generally explore evidence of information exchange between GPs and specialists	Bibliographic academic databases were searched for the period 1995 – 2011 (MEDLINE, Embase, CINAHL, and PSYC INFO Scius and Google Scholar. Org. websides)	In all 32 paper and the librarian also explored the gray literature using sources suggested by the Cochrane Collaboration, were searched	The study showed variation in the quality of communication between GPs and mental health specialist, although care management inevitably requires collaboration among many providers to meet patients need over time. Patient-centered care, such as explaining the purpose of a consultation request to the patient, need attention, being among the least investigated in the review literature
9: Gronholm et al., (2017) UK/Australia	Examine stigma related influences on pathways of care in each stage of the pathway in first episode psychosis	Review which included quantitative, qualitative and mixed methods studies from 1996 and 2016	CINAHL, EMBASE; Medline, PsycINFO were used. Data synthesis was conducted in three stages. First thematic analyze, was undertaken to synthesize the finding of articles reporting qualitative data. Second narrative synthesis, third stages involved a meta- synthesis	In all 40 studies. Both patients and health personnel views were included. People aged up to and including 40 years	Lack of information could result in increased perceived stigma. Patients also perceived devaluations by service providers. Perceived stigma reduction could decrease if treatment were normalized as sell as working with info to environment
10: Mutschler et al., (2019) Canada/USA/UK	To capture and consolidate the current understanding of the experiences of individuals post-discharge who are transitioning back into their communities	Systematic review. Both quantitative and qualitative design is included	Systematic literature search, following PRISMA guidelines. Using Medline, PsycINFO, Google Scholar, HealthStar Included 27 papers from a variety of countries	In all 27 paper were included. 18 quantitative and 9 qualitative papers. Only patient perspectives All papers lacking patients´ experiences were excluded	Themes identified as necessary for transition were patient safety, supported autonomy, and activities in the community. Barriers were poverty, interpersonal difficulties and stigma. All pointing to the need of targeting the identified challenges
11: Neame et al. (2019). UK	Explore the effects of implementation of health information technologies in care pathways	Systematic review with inclusion of 44 papers Systematic literature search, following PRISMA	Using Medline, EMBASE, CENTRAL. 94% reported from hospital care and treatment and 6% from community service	44 paper focused on health technology Electronic documentation. 16 were before-and-after studies,14 was noncomparative, 5 were interrupted time series studies, 4 were retrospective cohort studies, 2 were cluster randomized controlled trials, and there were 1 each of controlled before-after, prospective case–control, and prospective cohort studies	Some evidence that health information technology improving objectively measured patient outcome (mortality, patient-reported outcome measures, biochemical markers, and disease activity). More research is needed
12: Rotter et al (2010) Cochrane/ Germany	To assess the effect of clinical pathways on professional practice, patient outcomes, length of stay and hospital costs	Randomized controlled trials, controlled clinical trials, controlled before and after studies and interrupted time series studies comparing alone in clinical pathways with usual care as well as clinical pathways as part of a multifaceted intervention with usual care	Searched the Database of Abstracts and Reviews of effectiveness (DARE), The Effective Practice and Organization of Care Register, the Cochrane Central Register of Controlled Trials and bibliographic databases including MEDLINE, EMBASE, CINAHL, NHS EED and Global Health	In all 28 studies included. Two review authors independently screened all titles to assess eligibility and methodological quality. Studies were grouped into those comparing clinical pathways with usual care and those comparing clinical pathways as a part of a multifaceted intervention with usual care	Clinical pathways are associated with reduced in-hospital complications and improved documentation without negatively impacting on length of stay and hospital costs. (Author’s conclusions)
13: Storm et al., (2019) Norwegian /USA	Examine effective coordination between levels of care, challenges in providing continuity of care and quality of life in the transition process	Scoping review design followed Arkey & O`Malley´s (2005) five-stage framework	CINAHL; Cochrane, Medline, PsycInfo, Google scholar was used. Systematic review with inclusion of 16 papers with qualitative and quantitative design	In all 16 paper. Both patients and health personnel views were included. Paper from USA, Japan, UK. Individual with mental illness > 8 years old	Effective approaches addressed coordination challenges and resulted in better improvements in service utilization, social functioning and quality of life. Shared decision-making support for caregivers and addressing the challenges when patients are need of complicated medication regimes
14: Viggiano et al., (2012). USA	To provide an overview of current care transition intervention frameworks and models, and to identify components suited for more effectively managing transitions among persons with mental illness	A review of intervention models identified multiple models	PubMed, Google scholar, and grouped into two categories. 1.Models that have been put forward and tested in the area of medical care.2. Information about framework, conceptual models or descriptions of care transitions intervention	In all nine papers about transitions from hospital to outpatient care. Managing transitions among persons with SMI	A core set of nine care transitions intervention components can stimulate the development of interventions that address transitions in the mental health population more effectively
15: Vigod et al., (2013). UK	To describe and evaluate interventions applied during the transition from in-patient to out-patient care in preventing early psychiatric readmission	Systematic review of 15 paper about transitional interventions among adults admitted to hospital with mental illness where the study. outcome was psychiatric readmission	Medline, CINAHL, EMBASE, PsycINFO and the Cochrane Library	I all 15 papers about transition from in-patient to out-patient care in reducing early psychiatric readmission among adults with mental illness	Effective transitional intervention components are feasible and likely to be cost-effective Our results are consistent with the findings of a previous review of pre-discharge interventions in psychiatry
16: Volpe et al., (2015). Romania	Comparison of mental health pathways in 23 different countries around the world	Due to different instruments and gathering of data, the planned comparison could only be performed at a few variables. PRISMA	From MELINE, OVID, EMBASE, PSYCINFO. Reference list from 1986 to 2013 with majority of studies from 2005 and newer	A global perspective of 34 papers on psychiatric pathway. Both patients and health personnel views were included. Studies from different country and continent	Identified referral delay differences between countries with a range from Romania, Cuba, Bangladesh and Albania to 25 weeks before seeing a psychiatrist. Also, the role of a general practitioners could either decrease or increase the referral time. No direct comparison of data concerning the pathway to mental healthcare at the world level, is available yet

**Table 2:** Characteristics of articles with primary studies

Author and country of origin	Aims	Study design	Data Collection and measurements	Study sample	Results
a: Akehurst et al. (2018). UK	To understand what contextual influences, mechanism and outcomes affect the implementation and use of localized, online care pathways (Health Pathways) in primary and secondary care	Mixed-measures design The study methodology draws on the realist approach to evaluation, providing an explanatory analysis aimed at showing what works for whom, under what circumstances, in what respects and how in order to provide an in-depth understanding of an intervention and how it can be made to work most effectively	Quantitative data included number of page views and conditions viewed. Qualitative data from semi-structured interviews and focus groups were gathered over a 6-month period and analyzed using NVivo software	General practitioners, nurses, practice managers, hospital consultants and system leaders	Find that use of pathways increases over time. *Themes developed*: showing how online care pathways were used – leadership, pre-existing networks and relationships; development of systems and processes for care pathways, the use of online care pathways to support decision-making and referral, and availability of resources. *Inter-related themes*: contextual influences, mechanism, and outcomes. *Recommendation*: improved data collection processes to understand how and why there was variance in the use of pathways
b: Biringer et al. (2017) Norway	To assess health care personnel’s perceptions of the organization of care processes in the specialist service in Norway. A further goal was to examine whether the staff considered the organization to be better in the care processes that were standardized using clinical procedure compared to pathways without such procedures	Assessing health care personnel’s perceptions of the degree to which the organization was patient focused, how well the treatment for the patient groups was coordinated, how well communication with patient and family worked, how well the collaboration with primary care worked, and whether the standardization of care processes was followed up	Care Process Self-Evaluation tool (CPSET) was used to evaluate how interprofessional teams in the specialist health service about their experiences with the organization of the treatment for specific patient groups	Staff took part from a total of six somatic hospitals and six psychiatric units in western Norway Regional Health Authority, were asked to complete the CPSET. Analyzes were based on responses from 239 employees in 22 valid care processes (48 per cent response rate)	The CPSET in the sample was higher than comparable international figures. However, Norwegian employees considered the follow-up of the care processes and the collaboration with primary care to be proper than other dimensions of care organization. Care processes with a written clinical procedure were better organized than process without such standardization
c: Hasson-Ohayon et al. (2016) Israel	Gaining a better understanding of the transition phase from psychiatric hospitalization back to the community	Qualitative methodology and narrative analysis. The analytic approach was guided by the interpretative phenomenological approach	Semi-structured interviews, Focusing on the subjective experience of the transition from the hospital to the community	Personal life stories of 15 people diagnosed with schizophrenia who had just returned to the community following a psychiatric hospitalization	Revealed different characteristics of the transition phase. In addition to oscillation between feelings of strength and vitality to vulnerability and despair, participants reported specific factors included social, familial, employment and professional aspect. The results emphasize the non-linear nature of the transition process and the special challenges involved. Results also stress the importance of supportive relationships and work
d: Khandaker et al. (2013) UK	To evaluate how a model ‘payment by results’ for mental health works out in community mental health practice, including its impact on quality of patient care, mental health professionals and primary care	A theoretical sampling method was used to identify members of community care pathways involved in directly patient care (e.g. inpatient ward, crisis, and home treatment teams)	In total 19 interviews. Each participant took part in a private one-to-one in-depth face to-face interview at his/her own workplace lasting up to an hour. Recorded interviews were coded and analyzed thematically using grounded theory approach	Doctors, multidisciplinary staff and Trust managers in community and in acute care (e.g. inpatient ward, crisis and home treatment teams)	The model led to more focused interventions being offered and working in pathways was generally seen as a positive change; practitioners being held account over clear standards of care, more cost-effective and allows for active case management and clear clinical leadership. The arbitrary time frame, strict criteria and thresholds for different teams could create issues. Improved communication, flexible and patient-centered approach, staff supervision, and increasing support to primary care were felt to be central to this model working efficiently and effectively
e: Seys et al. (2017) Belgium	Care pathways are better perceived than care processes organized without care pathways. To evaluate the extent to which team scores correlate for care processes with or without care pathways	Multilevel analysis was used to compare care processes without and with care pathways. Almost all care processes were evaluated either before implementation of a care pathway, during the development phase or after implementation	The statistical analysis included 2692 questionnaires from 87 Belgian organization and 21 organization from Netherlands Care Process Self-Evaluation tool (CPSET) was used to evaluate how healthcare professionals perceived the organization of care processes	In all 108 organizations from Netherlands/Belgian; acute hospitals, psychiatric hospitals, specialized hospitals and primary care	A significant difference between care processes with and without care pathways was found. A care pathway in use led to significant better scores on the overall CPSET scale and subscales, ‘coordination of care’ and ‘follow-up of care’. Physicians had the highest score on the overall CPSET scale
f: Sather et al. (2016) Norway	Exploring community health personnel’s experiences of care pathways in patient transition between inpatient and community health services	A descriptive qualitative design was chosen	Four focus groups interviews were conducted. Interviews were analyzed thematically	Twelve health employees from 7 community health care settings shared their experiences (1 urban and 6 rural)	Main themes were identified: integrated care and patient activation. Promoting factors affecting smooth CP were identified for successful patient transition: opportunities for information sharing, implementation of systematic plans, use of e-messages, around-the clock care, designing one responsible health person in each system for each patient, and involvement of patients and their families. Barriers to impede the patient’ transition between levels of care: the lack of a single responsible person at each level, insufficient meetings, the absence of systematic plans, difficulties in identifying the right staff at different levels, delays in information sharing, and the complexity of welfare systems negatively affecting patient dignity
g: Sather et al (2018) Norway	Exploring community health personnel’s experiences of care pathways in patient transition between inpatient and community health services	A descriptive qualitative design was chosen	Four focus groups interviews were conducted. Interviews were analyzed thematically	Twelve health employees from 7 community health care settings shared their experiences (1 urban and 6 rural)	Main themes were identified: integrated care and patient activation. Promoting factors affecting smooth CP were identified for successful patient transition: opportunities for information sharing, implementation of systematic plans, use of e-messages, around-the clock care, designing one responsible health person in each system for each patient, and involvement of patients and their families. Barriers to impede the patient’ transition between levels of care: the lack of a single responsible person at each level, insufficient meetings, the absence of systematic plans, difficulties in identifying the right staff at different levels, delays in information sharing, and the complexity of welfare systems negatively affecting patient dignity
h: Sather et al. (2019) Norway	Exploring former patients’ views of pathways in transition between psychiatric hospitalization and the community	A descriptive qualitative design was chosen	Interviews from three focus groups were transcribed and analyzed thematically where themes describe promoting or inhibitory factors to the transition phase	Three focus group interviews with former patients were with a total of 10 informants from five different communities were conducted	Four main paired themes were identified: (a) patient participation versus paternalism and institutionalization, (b) patient-centered care versus interpreted as humiliation, (c) interprofessional collaboration or teamwork versus unsafe patient pathways in mental health services, and (d) sustainable integrated care versus fragmented, noncollaborative care
i: Steinacher et al. (2012) Germany	To determine whether the implementation of a pathway would improve diagnosis and treatment in conformity with published guidelines	Quantitative study with a prospective, controlled design (a two-year process)	Questionnaires (before and after pathways). Differences between patients in ward A and B and longitudinal (pre and post) between patient groups	In all 114 patients with schizophrenia in open, general psychiatric wards, where treatment pathways were implemented in two different ways	The patients reported less treatment satisfaction after the implementation of the pathways. They offered no explanation for their findings. No significant intergroup differences between groups in ward A and B were found
j: Teshager et al. (2020) Northern Ethiopia	To assess pathways to psychiatric care and factors associated with delayed help-seeking among patients with mental illness using the WHO Pathway Study Encounter Form	A cross-sectional study was used	Data were collected using face-to-face interview from patients with various diagnosis of mental illness	Participants who attend outpatient treatment during the study period were included in the study using consecutive sampling technique	Significant delay in seeking modern psychiatric treatment. Religious healers were first source of help, due to mental illness was supernatural causes. Stigma and lack of awareness about where treatment is available were barriers to seeking appropriate care
k: Van Houdt et al. (2013) Belgium	To assess the extent to which care pathways support or create elements of the multi-level framework necessary to improve care coordination across the primary-hospital care continuum	An in-dept analysis of five local community projects located in four different regions to determine whether the available empirical evidence supported or refuted the theoretical expectations from the multi-level framework	Data were gathered using mixed methods, included structured face-to-face interviews, participant observations, documentation, and a focus group. Multiple cases were analyzed performing a cross case synthesis to strengthen the results	Staff from five local community projects located in four different regions in Belgium (hospitals and home care)	The construction of a new and use of an existing structure had a positive effect on exchanging information, formulating and sharing goals, defining and knowing each other’s roles, expectations and competences and building qualitative relationships
l: Wright et al. (2015) UK	To explore the nature of service user involvement in the admission and discharge process into and out of acute inpatient mental health care	A qualitative study using focus group interviews were conducted winter 2013–2014	Focused on knowledge sharing at the points of transition of care in and out of inpatient mental health services in seven focus group interviews. A semi-structured interview-guide were used and lasted for approximately 60 min	Staff from acute, inpatient mental health ward, community staff and service user (total number of participants = 52)	Due to the lack of resources (inpatient beds and community care follow-up), the role service users could play was diminished. In their narratives, clinical staff associated the person with the process and used language which dehumanized the individuals

**Table 3:** General issues and factors

General issues	Factors (reference)
**Organizational issues/** Systems and procedures should be developed to ensure clear responsibilities and transparency at each stage of the pathways of care	Intervention on patient, provider and system levels stimulate transitions. (Viggiano et al. 2012) Seven successful components; facilitated pre discharge, post discharge and transition processes, and promoted timely communication of inpatient staff with outpatient care or community service providers after discharge. (Vigod et al. 2013) First contact is important. (Amaral et al. 2018) Information technology supported care pathways. (Neame et al. 2019) Care pathway in use led to significant better scores on the overall CPSET scale and subscale, ‘coordination of care’ and ‘follow-up care’ with primary care. (Seys et al. 2017) Specialist health service should improve the systematic follow-up of care pathways as well as the collaboration with primary care. (Biringer et al. 2017) Use of on-line evidence-based care pathways across primary and secondary care increased over time (Akehurst et al.2018) The care pathway led to more focused interventions being offered and the working in it were a positive change. (Khandaker et al. 2013) Systems and procedures should be developed to ensure clear responsibilities and transparency at each stage of the pathways of care. Around the clock care, designing one responsible health person in each system for each patient, and involvement of patient and their families. (Sather et al.2018) First contact was a physician, but referral source was emergency services. Ethnic determinants not in focus in CP. (Anderson et al. 2010) Patients emphasized the non-linear nature of the transition process. (Hasson-Ohayon et al.2016) Lack of integration between emergency, hospital, and community. (Amaral et al. 2018) Continuity challenges during transitions. (Storm et al. 2019)
**Resources and Outcomes/** Use of CP and information technology in improving objectively patient outcome	Positively impact on length of stay and hospital costs with CP. (Allen et al. 2009) More cost-effective care pathways and allows for active case management and clear clinical leadership. The care pathway led to more focused interventions being offered and working in it were a positive change. (Khandaker et al. 2013) Transitional intervention components are feasible and likely to be cost-effective. (Vigod et al. 2013) Care pathways showing promising results in increasing the quality and efficiency of care for patients diagnosed with schizophrenia. (Chen et al. 2015) Effective approaches addressed coordination challenges and resulted in better improvements in service utilization (Storm et al. 2019) Use of information technology improved objectively patient outcome. (Neame et al. 2019) (Akehurst et al. 2018)
**Information and Documentation/** Providing patients with enough information and structured, documented plans at the appropriate time	Care Pathways improve documentation. (Allen et al. 2009) (Rotter 2010) Found variation in the quality of written communication and information transfer between primary care and specialist mental health providers, and patient-centered care was among the least investigated topics. (Durbin et al. 2012) Structures had a positive effect on exchanging information, formulating, and exchanging information. (Van Houdt et al. 2013) Main barriers were communication errors. Adequate direct communication and proper documentation system between health personnel, patient participation in plans and working hour of ambulant teams were success factors. (Sather et al. 2016) Care pathways are useful for securing key objectives. Success with opportunities for information sharing, implementation of systematic plans, and use of e-messages. (Sather et al. 2016, 2018) Care processes with a written clinical procedure were better organized than processes without such standardization. (Biringer et al.2017) Themes developed with on-line care pathways showed how pathways were used: in leadership, relationships, support decision-making and referral, and available resources (Akehurst et al. 2018) Lack of information can result in increased perceived stigma (Gronholm et al.2017) Stigma and lack of awareness about where treatment is available were barriers to seeking appropriate care. (Teshager et al. 2020) Former patients reported shared decision making more precisely as *informed* shared decision, and that shared information between all parties is key. (Sather et al. 2019)
**Patient and Family’s Participation/** Continuous collaborative decision-making	Patients with two different wards reported less treatment satisfaction with clinical care pathways for schizophrenia after the implementation. (Steinacher et al. 2012) Due to the lack of resources (inpatient beds and community care follow-up), the role of service user could play was diminished. (Wright 2015) Patients revealed oscillation between feelings of strength and vitality to vulnerability and despair in transition phase. Patient emphasize the importance of supportive relationships and work. (Hasson-Ohayon et al. 2016) Families perceived care planning to be uncoordinated. Lived experienced were not appreciated. Collaborative decision-making not regularly experienced. Family involvement was found to be an important factor related to pathways to care communications constraints. (Doody et al. 2017) Care Pathways affected patient safety, supported autonomy and activities in community. (Mutchler et al. 2019) Improved information sharing in/between all care systems is imperative to strengthen patients` participation in decision making, ownership of the care plan and improve adherence to treatment. Patient participation in care plans a success factor. (Sather 2016, 2018,2019) Shared decision-making support for caregivers with complicated regimes (medication). (Storm 2019) Family involvement was found to be an important factor related to pathways to care. (Allan et al. 2020) There is a significant delay in seeking modern psychiatric treatment. Religious healers were first source of help for mental illness (Teshager et al. 2020)
**Clinical Care Issues and Teamwork/** Collaboration between mental health and other professionals to guarantee that planned activities meet patients’ needs	Care Pathways gave more interpersonal aspects, changing professional attitude positively. (Allen et al.2009) Patient-centered found that care was among the least investigated topics between CPs and mental health specialists. (Durbin et al. 2012) Support interprofessional teams in enhancing teamwork. (Deneckere et al. 2012) Practitioners being held account over clear standard of care. (Khandaker et al. 2013) Patients reported the formal professional support as important to their recovery process in general and in their transition to the community in particular. (Hasson-Ohayon et al. 2016) Shared decision-making support for caregivers with complicated regimes (medication). (Storm 2019) Patients participation in plans and working hours of ambulant teams were success factors. Key person handling all information and communication between levels of care. (Sæther et al. 2018) Regular meetings sharing key information; avoidance of delays extending inpatient status and block satisfactory transition to the community setting. (Sather et al. 2018) Mental health professionals, and general practitioners played a key role in help seeking. (Allan et al. 2020)
**Ethical Issues/** Respectful communication and patient-centered care to avoid humiliating the patients	Stigma and discrimination limited factors in delivery of care. (Volpe et al. 2015) Clinical staff used language which dehumanized the individuals. (Wright et al. 2015) Dilemma when patient and health personnel have different options on treatment. Respectful communication to avoid humiliating the patients. (Sæther et al. 2016) The complexity of welfare systems negatively affected patient dignity. (Sather et al. 2018) Poverty, interpersonal difficulties, and stigma were barriers. (2019 Mutchler) (Gronholm 2017) Stigma and lack of awareness where treatment is available were barriers to seeking appropriate care for patients with various diagnosis of mental illness. (Teshager et al. 2020)

#### Stage 5: Collating, summarizing, and reporting results

To achieve a thematic presentation of the results and avoid bias, the lead author and the two other researchers read and reviewed the included full-text articles. The result of each of the included articles were summarized in separate text description paragraphs by the lead author. The other researchers read the text descriptions and suggested edits when necessary. These summaries were used to identify challenges in care pathways in transitions from hospital to the community for people with mental health problems, including their family and caregivers. The lead author identified what types of approaches and interventions models in the research literature showed evidence and improved the quality and efficiency of care pathways of hospital-to-community transitions for people with mental illness. The research team then discussed the approaches and interventions model of the study results and the challenges, themes and approaches identified. We used the Critical Appraisal Skills program (2018) to assess the methodological quality of the qualitative studies. The tool contains ten questions and assesses quality in three domains: validity, presentation, and impact of study results. We used the Cochrane Collaboration Risk of Bias Tool [47] to evaluate the studies that included quantitative results. This is a six -domain tool with a total of seven items that assess selection (two item), performance (one item), detection (one item), attrition (one item), reporting (one item), and other sources of bias (one item). ‘The risk of bias was evaluated independently by all researchers (EWS, MFS, FV, VCI; PC) included the researchers who extracted the data. Discrepancies were resolved by discussions until a consensus was reached [47].

## Results

Among 28 selected studies: was 19 quantitative synthesis and nine qualitative syntheses.

### Characteristics of the Included Studies

The aims of the six (of 19) review articles with quantitative data were as follows: identify the effectiveness of care pathways in mental health [48] provide evidence to improve the quality and efficiency of special diagnostic groups [49] assess the effects of pathways on practice, patient outcomes, length of stay and hospital costs [50] provide an overview of care and identify components for more effective transitions [51] describe and evaluate interventions in the transition from inpatient to outpatient care [52] and compare mental health pathways in 23 different countries [53].

For nine of 16 articles with both quantitative data and qualitative data, the aims included: examining the duration of untreated psychosis [54] highlighting evidence for each pathway [55] determining the relation between effectiveness and teamwork [56] identifying families’ experiences [57] gathering evidence on the quality of information transfer between primary care and specialist health care [14] examining stigma-related influences on pathways [58] understanding patient experiences after discharge from hospitals to community health care services [59] exploring the effects of implementation of health information technologies in care pathways [60] and identifying effective coordination between levels of care and continuity in the transition process [3] and to synthesize the available literature on pathways to care in ‘At-Risk mental States/ARMS or prodromal psychosis [61].

Among the primary studies (12 articles), the aims for the three articles with quantitative data were as follows: assess personnel perceptions of care processes and examine whether staff consider the differences between pathways with standardized clinical procedures and pathways without such procedures [62] analyse whether care pathways lead to better organization of care processes [63] determine whether the implementation of a pathway improves diagnosis and treatment in conformity with published guidelines [64].

Of the twelve articles with qualitative data, the aims were as follows: understand the contextual influences, mechanisms and outcomes that affect the implementation of online pathways [13] evaluate how the ‘payment by result’ model works in community mental health, including its impact on the quality of patient care, staff and primary care [65] assess the extent to which pathways support or inform the creation of elements of frameworks to improve care coordination across the primary hospital care continuum [8] explore the nature of service user involvement in the admission and discharge processes of acute inpatient mental health care [66] gain a better understanding of the transition phase from psychiatric hospitalization back to community for people diagnosed with schizophrenia [67] identify patients and healthcare professionals challenges and barriers in the transitional process between primary to secondary mental health services [20, 22, 23] and determine whether implementation of a pathway would improve diagnosis and treatment in conformity with published guidelines [68].

The quality appraisal of the articles that included quantitative research methods indicated that the studies had a high risk of bias. One of three studies met all ten criteria suggested by Critical Appraisal Skills Programme (CASP). Other bias description’ was noted in 16 of 28 of the articles had implementation problems, with small sample size [47, 69].

### Care Pathways Challenges between Mental Health Services

There were six major themes identified in the articles related to the challenges and barriers of pathways in the transition from hospital to community**:** (a) Organization, (b) Resources, (c) Information and Documentation, (d) Patients and Families, (e) Clinical Care and Teamwork, and (f) Ethics.

### Organization

Research found that the first contact for patients with psychosis was a physician, but the referral source was emergency services [54]. Viggiano et al. [51], found that a core set of transition intervention components could stimulate the development of interventions at the patient, provider, and system levels. More effective transitions in mental health care pathways included special procedural guidelines and instructions and links to national guidelines provided in the transition phase; prehospital, hospital, outpatient, home [55]. Amaral et al. [55], indicated the importance of the first contact in pathways to mental health care, and that there is a lack of integration between emergency departments, hospitals, and community services. Hasson-Ohayon et al. [67], revealed different characteristics of the transition phase for people with schizophrenia who had just returned to the community following a psychiatric hospitalization. The article emphasized the non-linear nature of the transition process and the special challenges involved. Storm et al. [3], found continuity challenges in care during transitions and services for people with serious mental illness. Effective approaches addressed coordination challenges and resulted in improvements in service utilization, social functioning, and quality of life. Seys et al. [63], identified a significant difference between care processes with and without care pathways, for ‘coordination of care’ and ‘follow-up care’ in primary care. Biringer et al. [62], found that Norwegian employees considered follow-up care processes and collaboration with primary care to be poorer than the other dimensions of care organizations. Care processes with written clinical procedures were reported to be better organized than processes without such standardization. Sather et al. [22], exploring community health personnel’s experiences of care pathways in patient transition between inpatient and community mental health services, suggested that systems and procedures should be developed to ensure clear responsibilities and transparency at each stage of the pathway of care.

### Resources and outcomes

Allen et al. [48], found that there was a positive impact on length of stay and hospital costs with care pathways. Khandaker et al. [65], indicated that care pathways were effective and allowed for active case management and clear clinical leadership. The care pathway led to more focused interventions being offered. Vigod et al. [52], concluded that transitional intervention components are feasible and likely to be cost-effective. Facilitated pre-discharge, post-discharge, and transition processes; and promoted timely communication of inpatient staff with outpatient care or community service providers after discharge was successful components that reduced hospital readmission. Chen et al.^,^ [49] found that care pathways showed promising results in increasing the quality and efficiency of care for patients diagnosed with schizophrenia but that more evidence was needed. Akehurst et al. [13], concluded that the use of localized, online evidence-based care pathways across primary to secondary care increased over time and showed that care pathways were used in leadership, relationships, and networks to support decision making and referrals and provided information on the availability of resources. Neame et al. [60], found that health information technology supported care pathways and improved objectively measured patient outcomes.

### Information and documentation

Allen et al.^,^ [48] found that care pathways are effective with patients with predictable mental health symptoms. Care pathways improve documentation, communication, and change professionals’ behaviour positively. Rotter et al.^,^ [50] found that care pathways reduced in-hospital complications and improved documentation without negatively impact to the length of stay and hospital costs. Durbin et al.^,^ [14] focused on the content and/or timing of written communications and found variation in the quality of communication between care pathways and mental health specialists and that patient-centred care was among the least investigated topics. Research found that existing and new care pathways in four communities had positive effects on exchanging information; formulating and sharing goals; defining and knowing each other’s roles, expectations, and competences; and promoted the relationship between care pathways and care coordination [13]. Sather et al. [20], found that that clinical pathways are useful for securing key objectives at the interface between hospital and community based psychiatric care. Improved information sharing in/between all care systems is imperative to strengthen patients’ participation in decision-making, ownership of care planning and improving adherence to treatment. Adequate communication and proper documentation systems were factors in this success by avoiding communication errors that were the main barriers. Biringer et al. [58], found that care processes with a written clinical procedure were better organized than processes without such standardization. Gronholm et al. [58], identified themes related to the relationship between stigma and care pathways among people experiencing first-episode psychosis or at a clinically defined risk of developing psychotic disorder. The findings indicated that a lack of information could result in increased perceived stigma. Akehurst et al. [13], showed that care pathways were used to support decision making and referrals and provided information on the availability of resources. Sather et al. [23], emphasized that former patients reported shared decision making more precisely as *informed* shared decision making, and that shared information between all parties is key. Teshager et al. [68], found that stigma and lack of awareness about where treatment is available were barriers to seeking appropriate care.

### Patient and family’s participation

Steinacher et al. [63], tested the effects of clinical care pathways for schizophrenia in open general psychiatric wards with two different implementation strategies. The authors offered no explanation for their findings. Due to the lack of resources the role of the service user was diminished [66]. Patients revealed oscillation between feelings of strength and vitality to vulnerability and despair in the transition phase and emphasized the importance of supportive relationships and work [67]. Doody et al. [57], explore families’ experiences of engaging in care planning within adult mental health services. Families perceived that care planning was uncoordinated and that their lived experiences were not always appreciated; they did not regularly experience collaborative decision making but did experience communication constraints, protection of confidentiality and providers’ claims of ‘insider knowledge’ of service users. Sather et al. [20, 22, 23], suggested that improved information sharing in/between all care systems is imperative to strengthen patients’ participation in decision making, ownership of the care plan and improve adherence to treatment. Patient participation in sketching of care plans were a success factor. Storm et al. [3], emphasized that effective coordination of pathways of care resulted in better social functioning and quality of life. Shared decision-making support for caregivers was found to be important, especially when patients needed complicated medication regimes. Teshager et al. [68], found that there is significant delay in seeking modern psychiatric treatment with religious healers providing the first source of help for mental illness. Allan et al. [61], found that mental health professionals, and general practitioners played a key role in help seeking. Family involvement was also found to be an important factor.

### Clinical care and teamwork

Allen et al. [48], found that care pathways promoted interprofessional aspects of care and positively influenced professional attitudes. Durbin et al. [14], found that patient-centered care was among the least investigated topics between care pathways and mental health specialists. Deneckre et al.^,^ [56] revealed that care pathways have the potential to support interprofessional teams in enhancing teamwork. Khandaker et al. [65], found that a care pathway model for community mental health services led to more focused interventions being offered and implemented, resulting in positive changes; staff were also held accountable for clear standards of care. Arbitrary time frames, strict criteria and thresholds for different teams could create issues. Improved communication, a flexible and patient-centred approach, staff supervision, and increased support in primary care were felt to be central to this model working efficiently and effectively. Hasson-Ohayon et al. [67], reported the formal professional support as important to their recovery process in general and in their transition to the community, mostly associated with the need for continued care and having a therapeutic setting to attend. Sather et al. [20, 22], suggested that patients’ participation in plans and working hours of ambulant teams were success factors. A key person to handle all information and communication between levels of care was recommended.

### Ethical issues

Volpe et al. [53], found that the role of general practitioners could either decrease or increase the referral time to care pathways. Stigma and discrimination towards patients with mental illness are limiting factors for the equal delivery of mental healthcare. Wright et al. [66], focused on knowledge sharing at points of transition of care into and out of inpatient mental health services. The findings showed a loss of the voice of service users at key transition points. It was concluded that these encounters can have lasting negative effects, indicating the importance of ensuring that service users have a voice in determining what happens to them. Gronholm et al. [58], identified themes in relation to stigma on pathways to care among a target population and illustrates the complex way stigma-related processes can influence help-seeking and service contact among first-episode psychosis and at-risk groups. Lack of information could result in increased perceived stigma. Sather et al. [20, 22], exploring community health personnel experiences of care pathways in patient transition between inpatient and community mental health services, suggested respectful communication to avoid humiliating the patients. The complexity of welfare systems negatively affected patient dignity. Mutcheler et al. [59], identified themes related to transition, patient safety, supported autonomy, and activities in the community. Barriers were poverty, interpersonal difficulties, and stigma. Teshager et al. [68], also found that stigma and lack of awareness about where treatment is available were barriers to seeking appropriate care for patients with various diagnosis of mental illness.

## Discussion

The literature review identified themes and issues relating to: Organization; Resources and Outcomes; Information and Documentation; Patient and Family’s Participation; Clinical Care and Teamwork; and Ethics. Articles supporting the included studies are indicated with reference number in italics. Issue concerting the first of our themes, *organization*, seem to embrace our findings concerning improving pathways of care, and system and procedures should be developed to ensure clear responsibilities and transparency at each stage of the pathways of care. Effective transitional interventions at patient, provider and system levels were found to be feasible and likely to be cost-effective. To address transitions in the health mental population more effectively care pathways need special procedural guidelines, instructions and links to national guidelines provided in the prehospital, hospital, outpatient, and home phase [51]. A study of Sather [70] concluded that to achieve sustainable integrated care, pathways of care should also describe content of the transitional phase in and out of hospitals and community services.

Facilitated pre-discharge, post-discharge, and transition processes, and promoted timely communication of inpatient staff with outpatient care or community service providers after discharge were successful components [52]. First contact is important in pathways of mental health care, and there is lack of integration between emergency departments, hospitals, and community [55]. Care pathways led to better coordination of care and follow up with primary care [62]. The first contact for patients was a physician, but the referral source was emergency services. Ethnic determinants of the pathway, or the impact of the pathway to care on treatment delay was not found [54]. Earlier research has stated that the context of care pathways implemented in complex organizations must be considered in both external and internal contexts and have better descriptions of implementations and the contextual factors [12, 71]. An understanding of the development changes and implementation process of a particular context is critical to support multidisciplinary teams in their search for excellence; and it is recommended that clinicians and managers should evaluate each of their individual projects to ensure that patient and organizational outcomes are improved [72, 73]. Information technology supported care pathways and improved objectively measured patient *outcomes and resources* [13, 60]*.* It supports decision-making and referral, and available resources; made more cost-effective care pathways and allows for active case management and clear leadership, relationships, support decision-making and referral, and available resources [13, 59, 63]. It was found that care pathways may lead to better clinical outcomes, ensure more focused interventions and be valued by workforce [48, 52, 65]. This is in line with Mater et al. [74], who emphasized that development and implementation of care pathways are knowledge-based systems, care pathways optimize medical behaviour, and as clinical decision support systems, care pathways play a role in improving healthcare quality [74]. Our review revealed that pathways are a solution to safety problems and can improve extended care episodes as part of preventing unnecessary hospitalization [3, 59, 66]. Development and implementation of care pathways is labour-intensive; thus, resources should be optimally used.

Care pathways in mental health were found to be effective and improve *information and documentation*, providing patients with enough detail about their care and structured, documented plans at the appropriate time. Former mental health patients reported that shared information between all parties involved in care pathways is key. Proper documentation systems between health personnel, and patient participation in plans were success factors.

Opportunities for information sharing, implementation of systematic plans, use of e-messages were identified for successful patient transition, and the absence and systematic plans and delay in information sharing were barriers found to impede the patients’ transition between levels of care [20, 22, 23]. Care pathways were found to reduce in-hospital complications and improved documentation without negative impact on the length of stay and hospital cost [50]. This is in line with research in somatic health care [75, 76] that has shown that the implementation of a care pathway leads to increased or clearer documentation of care, and better interprofessional teamwork and better organized care [77, 78].

Continuous collaborative decision-making and *patient and family’s participation* was found to be an important factor related to pathways to care. Care pathways affected patient safety, supported autonomy and activities in community [59]. It was found that patients revealed oscillation between feelings of strength and vitality to vulnerability and despair in transition phase and emphasized supportive relationships and work [67]. Continuous collaborative decision making was emphasized, but this was not regularly experienced. Families perceived that care planning was uncoordinated and that their lived experiences were not always appreciated [57]. It was also found that improved information sharing in/between all care systems is imperative to strengthen patients’ participation in decision making, ownership of care plans and improve adherence to treatment. Additional, patient participation in care plans were success factor [20, 22, 23] as well as shared decision-making support for caregivers. Power and trust seem to be important factors that may increase as well as decrease patients’ dependency, particularly as information overload may increase uncertainty [79].

*Clinical care and teamwork* were found to be important in pathways of care; collaboration between mental health and other professionals was a guarantee that planned activities meet patients’ needs, and pathways gave more interprofessional aspects, changing professional attitude positively [48]. Regular meetings sharing key information and avoidance of delays that extend inpatient status and block satisfactory transition to the community setting are key [22]. It was revealed that care pathways have the potential to support interprofessional teams in enhancing teamwork. The most frequent positive effects were on staff knowledge, interprofessional documentation, team communication and team relations ^56^. Open communication, well organized and delegated coordinating roles for interprofessional care team delivery are essential elements so that the service is consistent with agreements reached with patients and relatives [24, 25].

Mental health professionals support is key in help seeking for patients and important to their recovery process in general alongside transition to the community [67]. Patient-centred care was among the least investigated topics between mental health specialists [14]. Research indicated that creating reliable treatment and care processes, a stimulating social climate in wards, and better staff-patient communication enhances patient perceptions of safety during inpatient care [79]. The review showed considerable variations in the *ethics* relating to mental health care pathways. The role of general practitioners could either decrease or increase the referral time, and stigma and discrimination towards patients with mental illness are limiting factors for the equal delivery of mental healthcare [53]. The results highlighted the disconnect that occurs for patients as they transition from hospitals back to their communities, indicating the need for effective, ethical transitional interventions that target these challenges [59]. Lack of information could result in increased perceived stigma and devaluation of people with mental health challenges. Stigma and lack of awareness where treatment is available were barriers to seeking appropriate care for patients with various diagnoses of mental illness. The complexity of welfare systems negatively affected patient dignity, with patients and health personnel viewing treatment options differently. Respectful communication to avoid humiliating the patients was emphasized [20, 66]. Without ownership in decision making, patients in psychiatric inpatient care settings may prove less treatment compliant [34]. A recent study suggested that greater epistemic justice might be achieved by shared decision-making processes in which patients are engaged as a full, collaborative partner in their care [35]. Many of the studies were characterized by small study samples, no randomization and lack of control group, which increases the risk of bias and the ability to draw conclusions about outcomes [47]. Despite a comprehensive literature search of multiple databases that used broad search terms, the search may have missed relevant studies. These searches did not result in the inclusion of additional studies. It is recommended to consult experts in the fields as a separate but optional stage in the search strategy [42, 45]. However, expert consultation was not feasible in this study. Discussions among the authors on the depth and breadth of the review during the study selection stage may have resulted in a reduction of the scope. An assessment of methodological quality of the studies is debated within the scoping review tradition (Pham et al.; [80]). In the present review, the quality assessment was performed to identify the strength of the evidence base and was not used as a tool for the exclusion of studies.

## Conclusion

In pathways of care, systems and procedures can ensure clear responsibilities and transparency. Information technology could support decision-making and referral to improve objectively patient outcomes in care pathways. Collaboration between mental health and other professionals can guarantee that planned activities meet patients’ needs through regular meetings sharing key information. Around-the-clock ambulant teams in the community are important alongside informed shared decision making, information and documentation between all parties to support patient participation. Respectful communication can avoid patient humiliation that could undermine treatment compliance. The combination of professional and patient perspectives best promotes positive outcomes from sustainable care pathways.

## Supplementary Information

**Additional file 1.**

## Data Availability

This study is a review study, and all raw data can be found in the articles which are published in our reference list in the end of our article. We don´t have any other raw materials.
