# Supplementary material for: Exploring sustainable care pathways - a scoping review
Source: BMC Health Serv Res. 2022 Dec 30;22:1595. doi: 10.1186/s12913-022-08863-w (PMC9801530; doi:10.1186/s12913-022-08863-w)
Supplement: Supplementary file 1 — Additional file 1. [file 12913_2022_8863_MOESM1_ESM.zip › 16.11.22 Exploring Sustainable Care Pathways PRISMA_2020_abstract_checklist.pdf]

PRISMA 2020 for Abstracts Checklist

Section and Topic	Item #	Checklist item	Reported (Yes/No)
**TITLE**			
Title	1	Exploring sustainable care pathways – A scoping review (Identify the report as a systematic review).	Yes
**BACKGROUND**			
Objectives	2	Patients with mental health problems experience numerous transitions into and out of hospital. The review studies assessing clinical care pathways between psychiatric hospitalization and community health services. (Provide an explicit statement of the main objective(s) or question(s) the review addresses.)	Yes
**METHODS**			
Eligibility criteria	3	Used publications between 2009 – 2020 to allow a broad scoping review of the published research. Sixteen review-articles were identified, 12 primary studies were chosen, both on care pathways in the transition between psychiatric hospital and community. The study population included adult individuals (18 years of age or older). Excluded studies which children were involved. (Specify the inclusion and exclusion criteria for the review).	Yes
Information sources	4	ProQuest/Health & Medicine, CINAHL Complete, Cochrane trials and Cochrane reviews, Psych Info, Medline, PubMed, and Google Scholar. The searches included studies published in English between 2009 and 2020. (Specify the information sources (e.g., databases, registers) used to identify studies and the date when each was last searched).	Yes
Risk of bias	5	We used the Critical Appraisal Skills program (2018) to assess the methodological quality of the quality studies (validity, presentation, and impact of study results). We used the Cochrane Collaboration Risk of Bias Tool to evaluate studies that included quantitative results; Six-domain tool assessing selection, performance, detection, attrition, reporting, and other sources of bias. (Specify the methods used to assess risk of bias in the included studies).	Yes
Synthesis of results	6	The study population included adults' individuals; people who are 18 years of age or older. We excluded studies which children were involved. Care pathways for specific mental health diagnoses were not searched for but included if they fitted the overall purpose of the study. We excluded editorials and discussion papers, and research protocols. (Specify the methods used to present and synthesise results).	Yes
**RESULTS**			
Included studies	7	Sixteen review-articles were identified, 12 primary studies were chosen, both on care pathways in the transition between psychiatric hospital and community. A table summarize relevant characteristics of the studies: Organizational issues/ /Resources and Outcomes/ Information and Documentation/Patient and Family's Participation/Clinical Care Issues and Teamwork/Ethical Issues. (Give the total number of included studies and participants and summarise relevant characteristics of studies).	Yes
Synthesis of results	8	Organizational issues: Systems and procedures to ensure clear responsibilities and transparency at each stage of the pathways of care. Resources: Information-technology in objectively improving patient outcome. Information/documentation: Providing patients with adequate structured information and documented plans at the appropriate time. Patient/families: Continuous collaborative decision-making. Clinical care and	Yes

Section and Topic	Item #	Checklist item	Reported (Yes/No)
		teamwork: Collaboration between mental health and other professionals to guarantee that planned activities meet patient need. Ethical issues: Respectful communication and patient-centred, non-humiliating care. All with references from authors chosen in the scoping review. (Present results for main outcomes, preferably indicating the number of included studies and participants for each. If meta-analysis was done, report the summary estimate and confidence/credible interval. If comparing groups, indicate the direction of the effect (i.e., which group is favoured).)	
**DISCUSSION**			
Limitations of evidence	9	We used the Critical Appraisal Skills program (2018) to assess the methodological quality of the quality studies (validity, presentation, and impact of study results), and used the Cochrane Collaboration Risk of Bias Tool to evaluate studies that included quantitative results; Six-domain tool assessing selection, performance, detection, attrition, reporting, and other sources of bias. Many of the studies were characterized by small study samples, no randomization and lack of control group, which increases the risk of bias and the ability to draw conclusions about outcomes. (Provide a brief summary of the limitations of the evidence included in the review (e.g., study risk of bias, inconsistency and imprecision).)	Yes
Interpretation	10	System and procedures ensure clear responsibilities and transparency. Information technology support decision-making and referral and objectively improve patient outcomes in care pathways. Collaboration between mental health and other professionals guarantee that planned activities meet patients' needs along with regular meetings sharing key information. Around-the-clock ambulant-teams important to transition success. Informed-shared decision-making between parties, support patient participation and respectful communication. (Provide a general interpretation of the results and important implications).	Yes
**OTHER**			
Funding	11	This study was funded by the Norwegian University of Technology and Science (NTNU). (Specify the primary source of funding for the review).	Yes
Registration	12	Review studies have no need for application to ethical committees in Norway because no patients/respondents are needed for these studies. The included studies all had statements on ethics. No registration number is used for this study. (Provide the register name and registration number).	Yes

From: Page MJ, McKenzie JE, Bossuyt PM, Boutron I, Hoffmann TC, Mulrow CD, et al. The PRISMA 2020 statement: an updated guideline for reporting systematic reviews. BMJ 2021;372:n71. doi: 10.1136/bmj.n71
