# Supplementary material for: Exploring sustainable care pathways - a scoping review
Source: BMC Health Serv Res. 2022 Dec 30;22:1595. doi: 10.1186/s12913-022-08863-w (PMC9801530; doi:10.1186/s12913-022-08863-w)
Supplement: Supplementary file 1 — Additional file 1. [file 12913_2022_8863_MOESM1_ESM.zip › 16.11.22 Exploring Sustainable Care Pathways PRISMA_2020_checklist.pdf]

PRISMA 2020 Checklist

Section and Topic	Item #	Checklist item	Location where item is reported
**TITLE**			
Title	1	Exploring sustainable care pathways – A scoping review (Identify the report as a systematic review).	Page1 and 2
**ABSTRACT**			
Abstract	2	See the PRISMA 2020 for Abstracts checklist.	Own list
**INTRODUCTION**			
Rationale	3	Patients with mental health problems experience numerous transitions into and out of hospital. (Describe the rationale for the review in the context of existing knowledge).	Pages 2-5
Objectives	4	Assessing clinical care pathways between psychiatric hospitalization and community health services. (Provide an explicit statement of the objective(s) or question(s) the review addresses).	Pages 2-5
**METHODS**			
Eligibility criteria	5	The study population included adult individuals (18 years of age or older). Excluded studies which children were involved. Used publications between 2009 – 2020 to allow a broad scoping review of the published research. (Specify the inclusion and exclusion criteria for the review and how studies were grouped for the syntheses).	Pages 6-8
Information sources	6	ProQuest/Health & Medicine, CINAHL Complete, Cochrane trials and Cochrane reviews, Psych Info, Medline, PubMed, and Google Scholar. The searches included studies published in English between 2009 and 2020. We did hand-search in key-journals, especially in articles in Norwegian Journals, that could not be accessed in full text in English. This were excluded. (Specify all databases, registers, websites, organisations, reference lists and other sources searched or consulted to identify studies. Specify the date when each source was last searched or consulted).	Pages 6-8
Search strategy	7	Following search terms were included to represent care pathways in mental health in transition from hospital to the community: Medical Subject Heading (MeSH) terms used for searches in Medline: care pathways, integrated care pathways, critical pathways, clinical pathways, mental health, adults, combined with 'transitions from hospital to community', 'referral', 'discharge', 'care planning', 'coordinating', 'hospital and mental health services'.(Present the full search strategies for all databases, registers and websites, including any filters and limits used).	Pages 6-8
Selection process	8	A total record was identified by the librarian conducting the systematic literature search and were imported into reference management software. After the removal of 200 abstracts, 28 full-text articles were assessed for eligibility, and then full-text articles excluded with reasons, we had at last 28 studies; studies included in qualitative synthesis 9 and studies included in quantitative synthesis 19. Two authors received the same first, and then two other authors received the half (of selected) 200. Disagreement regarding inclusion or exclusion were revolved through discussion. Four authors read the full text of the remaining 26 records and assessed their eligibility. The four authors conducted meetings to discuss their assessments of the abstracts and full-text articles and the inclusion and exclusion criteria to reach agreement on the studies to be included.(Specify the methods used to decide whether a study met the inclusion criteria of the review, including how many reviewers screened each record and each report retrieved, whether they worked independently, and if applicable, details of automation tools used in the process).	Pages 6-8
Data collection process	9	We extracted and coded each eligible and included article according to the following descriptive content of the 28 selected studies: 19 quantitative synthesis and nine qualitative syntheses. The descriptive data comprised authors, country of origin, aims, data collection and measurements, study sample and results. The extraction and charting of the data were conducted by author with input from two other researchers. (Specify the methods used to collect data from reports, including how many reviewers collected data from each report, whether they worked independently, any processes for obtaining or confirming data from study investigators, and if applicable, details of automation tools used in the process).	Pages 6-8
Data items	10a	Descriptive content is presented in table 1 and 2. Table 1 Characteristics of review articles, and Table 2 Characteristics of articles with primary studies are both listed as: Author and country of origin, Aims, Study design, Data Collection and measurements, Study sample, Results.<br>(List and define all outcomes for which data were sought. Specify whether all results that were compatible with each outcome domain in each study were sought (e.g., for all measures, time points, analyses), and if not, the methods used to decide which results to collect.	Pages 6-8

PRISMA 2020 Checklist

Section and Topic	Item #	Checklist item	Location where item is reported
	10b	See above, we have no more comments here. (List and define all other variables for which data were sought (e.g., participant and intervention characteristics, funding sources). Describe any assumptions made about any missing or unclear information).	
Study risk of bias assessment	11	This is partly described in tables at (the last two pages in the manuscript). (Specify the methods used to assess risk of bias in the included studies, including details of the tool(s) used, how many reviewers assessed each study and whether they worked independently, and if applicable, details of automation tools used in the process).	Page 19,20
Effect measures	12	Specify for each outcome the effect measure(s) (e.g., risk ratio, mean difference) used in the synthesis or presentation of results.	
Synthesis methods	13a	Our inclusion – and exclusion criteria for the study population included adults' individuals; people who are 18 years of age or older. We excluded studies which children were involved. And these criteria decided if the articles were compatible with enlightening our topics. (Describe the processes used to decide which studies were eligible for each synthesis (e.g., tabulating the study intervention characteristics and comparing against the planned groups for each synthesis (item #5)).)	Page 7,8
	13b	The qualitative articles did not describe missing respondents, this could of course initiate bias as well as in the quantitative articles. See item 18. (Describe any methods required to prepare the data for presentation or synthesis, such as handling of missing summary statistics, or data conversions).	See item 18.
	13c	This is described in Table 1 and Table 2. (Describe any methods used to tabulate or visually display results of individual studies and syntheses).	Page 10
	13d	This is described in Table 3. (Describe any methods used to synthesize results and provide a rationale for the choice(s). If meta-analysis was performed, describe the model(s), method(s) to identify the presence and extent of statistical heterogeneity, and software package(s) used.)	Page 10
	13e	The differences and similarities are described in Table 1, Table 2 and Table 3. (Describe any methods used to explore possible causes of heterogeneity among study results (e.g., subgroup analysis, meta-regression).)	Pages 10
	13f	Sensitivity analysis not done. (Describe any sensitivity analyses conducted to assess robustness of the synthesized results.)	
Reporting bias assessment	14	Confidence in the results in the included articles is based on thorough reading/analysis and long clinical experience. (Describe any methods used to assess risk of bias due to missing results in a synthesis (arising from reporting biases).)	
Certainty assessment	15	(Describe any methods used to assess certainty (or confidence) in the body of evidence for an outcome). Checklist- CASP	Pages 19-20
**RESULTS**			
Study selection	16a	This stage entailed the study selection process as illustrated in a Prisma flow diagram (Fig.1). The 283 articles in the scoping review resulted in 28 eligible full text articles. See item 8 above. (Describe the results of the search and selection process, from the number of records identified in the search to the number of studies included in the review, ideally using a flow diagram.)	Page 7
	16b	The study population included adults' individuals; people who are 18 years of age or older. We excluded studies which children were involved. Care pathways for specific mental health diagnoses were nor searched for but included if they fitted the overall purpose of the study. We excluded editorials and discussion papers, and research protocols. (Cite studies that might appear to meet the inclusion criteria, but which were excluded, and explain why they were excluded.)	Pages 6,8
Study characteristics	17	Table 3 Pathway Challenges listed as: 1) General issues and 2) Factors (reference): Organizational issues/ /Resources and Outcomes/ Information and Documentation/Patient and Family's Participation/Clinical Care Issues and Teamwork/Ethical Issues/ all with references from authors chosen in the scoping review.<br>(Cite each included study and present its characteristics.)	Page 10
Risk of bias in studies	18	We used the Critical Appraisal Skills program (2018) to assess the methodological quality of the quality studies (validity, presentation, and impact of study results). We used the Cochrane Collaboration Risk of Bias Tool to evaluate studies that included quantitative results; Six-domain tool assessing selection, performance, detection, attrition, reporting, and other sources of bias. (Present assessments of risk of bias for each included study).	Pages 8,9

PRISMA 2020 Checklist

Section and Topic	Item #	Checklist item	Location where item is reported
Results of individual studies	19	(For all outcomes, present, for each study: (a) summary statistics for each group (where appropriate) and (b) an effect estimates and its precision (e.g. confidence/credible interval), ideally using structured tables or plots.) Not appropriate	
Results of syntheses	20a	(For each synthesis, briefly summarise the characteristics and risk of bias among contributing studies.) See CASP-tables	Pages 19-20
	20b	(Present results of all statistical syntheses conducted. If meta-analysis was done, present for each the summary estimate and its precision (e.g., confidence/credible interval) and measures of statistical heterogeneity. If comparing groups, describe the direction of the effect.) Not appropriate	
	20c	(Present results of all investigations of possible causes of heterogeneity among study results.) See CRASP- table	Pages 19-20
	20d	(Present results of all sensitivity analyses conducted to assess the robustness of the synthesized results). Not appropriate	
Reporting biases	21	(Present assessments of risk of bias due to missing results (arising from reporting biases) for each synthesis assessed.) See CASP-table	Pages 19-20
Certainty of evidence	22	(Present assessments of certainty (or confidence) in the body of evidence for each outcome assessed.) See CASP-table	Pages 19-20
**DISCUSSION**			
Discussion	23a	The quality of articles was assessed based on the clarity of the research question, the data collection method used, the type and applicability of the qualitative analysis, the validity of the findings, and relevance of the results and conclusions. (Provide a general interpretation of the results in the context of other evidence.)	Page 18
	23b	Many of the studies were characterized by small study samples, no randomization and lack of control group, which increases the risk of bias and the ability to draw conclusions about outcomes. (Discuss any limitations of the evidence included in the review.)	Page 18
	23c	Despite a comprehensive literature search of multiple databases that used broad search terms, the search may have missed relevant studies. (Discuss any limitations of the review processes used.)	Page 18
	23d	The transition between health systems seems to be a critical phase in the total pathway of patient care. Therefore, there is a need for monitoring and identify what systems can be organized in better ways to provide patients with seamless and coherent transitions. We recommended further longitudinal research to investigate trends in patient involvement and participation in developing enhanced, well-organized transitions and specifically to determine best practices for shared interprofessional working according to pathway of care standards. (Discuss implications of the results for practice, policy, and future research).	Page 3,18
**OTHER INFORMATION**			
Registration and protocol	24a	Review studies have no need for application to ethical committees in Norway because no patients/respondents are needed for these studies. The included studies all had statements on ethics. No registration number is used for this study. (Provide registration information for the review, including register name and registration number, or state that the review was not registered.)	
	24b	The protocol mostly described the time schedule and digital meetings within the research group where search strategies were the most discussed topic. (Indicate where the review protocol can be accessed, or state that a protocol was not prepared.)	
	24c	No amendments in the in information and registration in the protocol. (Describe and explain any amendments to information provided at registration or in the protocol.)	
Support	25	This study was funded by the Norwegian University of Technology and Science (NTNU). (Describe sources of financial or non-financial support for the review, and the role of the funders or sponsors in the review.)	
Competing interests	26	There is not found any competing interests of this review authors. (Declare any competing interests of review authors.)	
Availability of	27	Table 1,2 and 3, show this in our article. (Report which of the following are publicly available and where they can be found template data	

PRISMA 2020 Checklist

Section and Topic	Item #	Checklist item	Location where item is reported
data, code and other materials		collection forms; data extracted from included studies; data used for all analyses; analytic code; any other materials used in the review.)	

From: Page MJ, McKenzie JE, Bossuyt PM, Boutron I, Hoffmann TC, Mulrow CD, et al. The PRISMA 2020 statement: an updated guideline for reporting systematic reviews. BMJ 2021;372:n71. doi: 10.1136/bmj.n71
For more information, visit: http://www.prisma-statement.org/
